# Utilizing machine learning algorithms for task allocation in distributed agile software development

**DOI:** 10.1016/j.heliyon.2024.e39926

**Published:** 2024-10-29

**Authors:** Dimah Al-Fraihat, Yousef Sharrab, Abdel-Rahman Al-Ghuwairi, Hamza Alzabut, Malik Beshara, Abdulmohsen Algarni

**Affiliations:** a Department of Software Engineering, Faculty of Information Technology, Isra University, 11622, Amman, Jordan; b Department of Data Science and Artificial Intelligence, Faculty of Information Technology, Isra University, 11622, Amman, Jordan; c Department of Software Engineering, Faculty of Prince Al-Hussien bin Abdullah for IT, The Hashemite University, 13133, Zarqa, Jordan; d Department of Computer Science, King Khalid University, Abha, 61421, Saudi Arabia

**Keywords:** Software engineering, Distributed agile software development, DASD, Task allocation, Machine learning (ML), Software project management

## Abstract

Distributed agile software development (DASD) has become a prominent software development approach. Proper task allocation is crucial in DASD to avoid undesirable outcomes including project rejection by clients, unfavorable team attitudes, and project failure. Coordination and communication issues occur as businesses embrace the DASD environment more frequently to tap into global talent and knowledge while cutting development expenses. To overcome these challenges, efficient task allocation planning becomes a crucial success component in software project management. The purpose of this study is to utilize machine learning (ML) predictive algorithms to determine the most appropriate role for a given task, with the aim of assisting software managers in making task assignments more efficiently and effectively in DASD environment. Preprocessing steps applied to the dataset include data cleaning, normalization, and partitioning into training, validation, and test sets. Four model classifiers were used in the experiment: Random Forest, Decision Tree, K-Nearest Neighbors (K-NN), and AdaBoost. The results showed that Random Forest outperformed the other classifiers in task allocation prediction, achieving an accuracy of 96.7 %, followed by K-NN (94.2 %), Decision Tree (93.5 %), and AdaBoost (93 %). The study demonstrates that ML models are effective in tackling task allocation issues in DASD settings, and the outcomes are promising.

## 1 Introduction

Sequential phases are used in traditional software development to create the software. It proceeds logically from requirement analysis to design and implementation to coding, testing, and maintenance; each phase must be finished before proceeding to the next. This method has been used for years due to its simplicity, methodical nature, and structure. Traditional approaches' main drawbacks include the need for a comprehensive list of requirements to start modifications when market conditions or organization ideals change throughout development [1].

Iterative and incremental development approach is the method used in DASD [2]. It places a strong emphasis on communication, flexibility, and regular feedback. With continuous integration and continuous delivery, development is carried out in brief iterations referred to as sprints [2]. ASD, Scrum, XP, and Crystal are all popular agile methodologies. The focus of agile software development is on prioritizing people and communication over tools and procedures. Delivering functional software is prioritized above producing comprehensive documentation, and customer involvement is favored over strict contract negotiation [3]. It is preferred to embrace change and be adaptable rather than rigorously follow a predefined strategy. Agile development emphasizes face-to-face communication as a key component, and team success is measured by the creation of functional sub-modules that are delivered to the client at the end of each sprint [4]. The phases of DASD include pre-project planning, the sprinting phase, the development phase, and the final release phase [2].

In a distributed context, multiple teams work together on the same project while being dispersed across various locations in the world. These teams might include people of various racial backgrounds, religious beliefs, moral standards, work ethics, and degrees of drive. They all adhere to agile software development standards and maintain regular interactions with project stakeholders to obtain feedback and input, despite their differences, contributing to the project's success [5]. For this, efficient channels of communication are used, allowing for the necessary modifications to the development processes depending on daily input [6]. The final products are released after these phases are complete. Working remotely with agile methodology makes managing team members and backlogs a key effort. To guarantee successful project completion and on-time delivery, these factors must be managed properly [2,5].

In any project, task allocation is crucial to success, and in a dispersed environment, its importance is amplified [4]. It's critical to effectively allocate tasks by combining the proper responsibilities with the relevant skills in order to maximize production and meet project milestones on schedule. There are typically three ways to distribute work within a team:

- Ad hoc allocation refers to the process of distributing jobs as they come up, without a set framework or strategy. This technique allows for an approach, to managing teams, where individual responsibilities are assigned based on the skills and availability of each team member. It works effectively for projects involving teams [7,8].
-Dedicated developer allocation; with this method each task or user story is assigned to a developer. This allows team members to specialize in their areas and promotes a sense of accountability for their duties. This approach is particularly suitable for activities and projects that require knowledge and high levels of proficiency [7,8].
-Swarm based task allocation is a flexible approach that prioritizes collaboration over assigning tasks to specific individuals. The entire team works together as an unit leveraging their knowledge and perspectives on all aspects of the project. This strategy encourages collaboration, among team members and fosters a shared understanding of the project [7,8].

Due to geographical distances, different time zones, and potential linguistic obstacles, task assignment becomes even more challenging while working in a remote workplace. In these circumstances, task distribution is made easier with the usage of collaboration and communication tools. To achieve an equitable and effective task distribution, project managers and team leads must carefully take into account team members' talents, availability, and preferences [8]. The project's scope, the size of the team, and the intended level of collaboration all play a role in choosing the best task allocation technique. Choosing the appropriate strategy has a significant impact on project success. On the other hand, selecting an inappropriate approach could result in inefficiencies, delays, and a general reduction in project performance. Poor task allocation can lead to longer task completion times, lower-quality software, and probable cost and budget overruns [9].

Machine learning (ML) algorithms provide prediction-based approach without human involvement, and have made great strides and shown promising results in a variety of industries [[10], [11], [12]]. In this study, we utilized a real-world dataset and supervised ML algorithms to predict staff responsibilities in the setting of DASD environment. We assessed the performance and evaluated the accuracy and effectiveness of the supervised learning algorithms: Random Forest, AdaBoost, Decision Tree, and K-Nearest Neighbors (K-NN) for task allocation. The algorithms used have demonstrated their applicability and efficiency in task allocation within the DASD environment.

Our research provides valuable insights into the operational dynamics of distributed software development teams, particularly concerning project management. Consider an organization that has teams collaborating on various software projects, and each project has a designated project manager and a product owner. When the product owner introduces a new task which needs to be incorporated into the next development sprint, the project manager faces the challenge of selecting the most appropriate role for this task. By employing machine learning classifiers, our research illustrates how companies can accurately predict suitable role for specific tasks, thereby streamlining the allocation of responsibilities and enhancing overall productivity within the team.

The rest of this research is organized as follows: a summary of earlier relevant research on task allocation and ML applications in software development is presented in Section 2. The background information on machine learning algorithms is provided in Section 3. The processes used for developing our prediction model are described in Section 4, together with information about the dataset and assessment criteria used to judge the predicted performance. The results of the experiments are shown in Section 5, and discussion of results is presented in Section 6. The paper concludes in Section 7 with a summary of the main conclusions, and a list of possible directions for further study.

## 2 Related work

Task allocation remains a prominent research focus within agile software development. Aslam and Ijaz [13], for instance, introduced theoretical models aimed at optimizing task distribution by considering factors such as team members' technical skills, areas of expertise, and existing workload. Similarly, Yadav et al. [14] introduced a two-stage expertise scoring system for managing bug triage. Their approach involves evaluating various factors such as developers' skills, average time to resolve bugs, versatility, and the bug's priority.

Anvik et al. [15] developed a semi-automated method designed to simplify part of the bug assignment process, specifically the task of allocating reports to developers. Their method utilizes a machine learning algorithm to analyze the bug repository and learn which types of reports each developer typically handles. When a new report is submitted, the algorithm suggests a shortlist of developers who are well-suited to address it. This approach has achieved precision rates of 57 % and 64 % for the Eclipse and Firefox projects, respectively.

Nundlall and Nagowah [16] conducted a systematic review of previous research on task allocation and coordination in distributed agile software development. Their goal was to discover flaws in current agile practices in distributed teams and analyze the factors and dependencies that stymie project development. The review covered numerous methodologies, problems, causes, and dependencies affecting task allocation and coordination processes at various stages of agile software development. The findings underlined the rising complexity and time-consuming nature of task coordination as teams increase in distributed environments, particularly in large software organizations with distributed teams.

Aslam and Ijaz [17] present a framework for distributing tasks in agile software development to improve task allocation and project management by identifying factors and dependencies that influence task allocation decisions. The framework is divided into two stages: first, identifying the elements that influence task allocation, and second, utilizing a quantitative approach to assign tasks to team members based on their best fit with the criteria. These requirements take into account abilities, personal characteristics, and environmental factors. With this method, project managers have the ability to assess the quality of task assignments prior to, during, and after project finalization to reduce the risks that come with working in different settings.

Ijaz et al. [18] conducted a study that explores the challenges of distributing work in software development. The research emphasizes the importance of task allocation in achieving project outcomes and proposes solutions to address these challenges. The paper also highlights the advantages of implementing the suggested solutions, such as decision making, use of resources and reduced project failures. The proposed method for task allocation aims to support task distribution in distributed software development. It examines aspects of the task allocation context to identify requirements for decision making. Based on this information, the technique outlines tasks that should be performed to facilitate task allocation decisions. The evaluation of this technique focuses on these activities with the objective of establishing an approach, to task allocation in a distributed software development environment. This technique covers both level and low level processes by identifying, categorizing, scheduling and recommending actions that contribute to task allocation in a distributed agile environment.

Nundlall and Nagowah [19] used an approach called OntoDASD which aims to improve the coordination and task allocation in DASD. OntoDASD utilizes an ontology based method that captures factors and interdependencies required for task distribution and coordination among teams in a distributed setting. The aim of this approach is improving team coordination by encouraging communication and appropriate task allocation through concepts for sharing knowledge. By providing a representation of knowledge related to task allocation and coordination, this method effectively addresses challenges faced in DASD.

In the research conducted by Shafiq et al. [20] they introduced an approach known as TaskAllocator. This method aims to enhance the efficiency of task allocation in software development projects by focusing on team roles than members. By predicting roles for tasks, TaskAllocator improved the overall task allocation process, particularly in situations where developers are overwhelmed or relocated. The researchers evaluated the performance of TaskAllocator through ten case study projects in a real-world setting. Compared it with machine learning models. To assess its effectiveness, they integrated Task Allocator with the project management tool JIRA. Their approach utilizes short term memory (LSTM) for text classification tasks. By analyzing task allocations and their textual characteristics, TaskAllocator offers predictions for assigning roles to incoming tasks with an accuracy of 69 %.

In their research, William et al. [21] introduced an approach to allocate tasks in DASD environments using unsupervised learning to overcome the obstacles of language and cultural differences among team members. Their method involves employing a classifier-based prediction model to assess and prioritize employees based on their suitability for task assignments. The model functions by clustering and extracting topics from the input data, training initial classifiers, creating a new dataset, and then training a second-level classifier. The model offers several advantages over other task allocation methods, including improved accuracy and more effective suggestions for task assignments.

Reviewing the literature reveals that proper task assignment requires careful evaluation of a plethora of contributing aspects including personal, organizational, and environmental factors to improve the task allocation process. Most research in this domain has concentrated on coordination processes in agile or distributed contexts, with little emphasis paid to aspects that consider task allocation to improving decision making [2,3]. Additionally, previous research that has explored work allocation methods, human involvement remains a key component in the task allocation process such as ad-hoc, with a specific developer, or through task swarming, which is considered a shortcoming as it can lead to mistakes in task assignment and could threaten the project's viability [7,8]. Furthermore, the results achieved by previous research that explored semi-automated approaches for bug assignment using machine learning algorithms have been mixed. Notably, the precision rates of 57 % and 64 % achieved by Ref. [15] indicate that these methods may not be as effective as desired. Moreover, the application of these approaches to open-source projects yielded even less favorable outcomes [20]. These findings underscore the need for more robust and adaptable methods to enhance decision-making and improve accuracy for task allocation without human intervention [3,4,7,8,22]. Our study seeks to overcome these limitations by enhancing task allocation in a DASD environment within open-source development projects. We employed several supervised learning methods for this purpose, specifically evaluating four algorithms: Random Forest, AdaBoost, Decision Tree, and K-Nearest Neighbors. By comparing these methods, we demonstrate and compare their accuracy and effectiveness in task allocation. The prediction of outcomes is based on input data by identifying patterns and relationships within labeled training examples. The subsequent section provides an overview of the machine learning algorithms utilized in this study.

## 3 Machine learning algorithms

Supervised machine learning algorithms are employed to estimate output values from input data by constructing a function that reflects the relationships and patterns in labeled training data [23]. An overview of the selected ML algorithms is briefly described as follows.

• Random Forest (RF) is a classifier that stands out for its ability to generate multiple decision trees. Each tree is built using a selected subset of training samples and variables [24]. The main idea behind this approach is to combine the predictions from decision trees, which improves the overall accuracy and robustness of the classifier. By utilizing subsets of data and features during tree construction Random Forest reduces the risk of overfitting while enhancing the model's ability to generalize. This makes RF well suited for handling datasets with dimensional feature spaces while also minimizing the impact of noisy or irrelevant features. Moreover, RF has gained popularity in ML and data mining due to its computation. This is because the individual trees are trained in parallel allowing for processing.
•AdaBoost, also known as Adaptive Boosting, is a meta-algorithm used for classification. It combines the outputs of learners (other learning algorithms) to create a strong and accurate boosted classifier. Each weak learner focuses on aspects of the data. Their predictions are weighted and combined to form the final output of the boosted classifier. AdaBoost is commonly employed for classification tasks. Can also handle multiple classes or bounded intervals on the real line. One key advantage of AdaBoost is its ability to enhance classifiers by assigning weights, to misclassified instances and refining classification boundaries progressively. This boosting process significantly improves accuracy and robustness making AdaBoost widely utilized in machine learning applications [25].
•Decision Trees (DT) are learning techniques extensively used in data mining and machine learning. Decision trees utilize attributes or characteristics of data elements as paths towards reaching a desired outcome, which is represented by the end points of the tree [26]. The decision points have paths each corresponding to values or categories of the evaluated attribute. Decision trees provide an understandable representation of decision-making processes making them valuable for model predictions. They can handle types of data including numerical attributes and are especially useful in complex decision-making scenarios by capturing nonlinear relationships and interactions between attributes. However, it's important to address the issue of overfitting by employing pruning techniques or utilizing methods like Random Forest to improve performance and generalization capabilities [26]. Despite this limitation, decision trees remain an interpretable tool for making decisions and predicting outcomes across domains
•K- Nearest Neighbors (KNN) is a supervised learning approach used for classification and regression tasks [27]. It relies on identifying the k data points in the training set to make predictions for unseen data points. In classification tasks KNN assigns the majority class label among the k neighbors to the data point. In regression tasks it calculates the value of target variables, among the k neighbors as its prediction. The KNN algorithm is considered nonparametric which means it doesn't make any assumptions about the distribution of the data [27]. Its effectiveness relies on selecting the hyperparameter k. KNN is considered a straightforward and uncomplicated tool for datasets and situations where interpretability is crucial.

## 4 Research methods

The ML models employed in this study include Random Forest, AdaBoost, Decision Tree, and k-Nearest Neighbors. These models were selected due to their potential effectiveness in predicting roles for a given task in a distributed agile environment, a context that involves dynamic and collaborative team structures. Previous studies have demonstrated successful utilization of these models in classification tasks similar to ours, particularly in areas like bug triaging, smell detection, and role assignment [4,17,28]. We utilized Bag of Words (BoW) approach for term-frequency to encode the task titles and relevant textual data as features, which were subsequently applied during the training phase.

In our research, the focus was on how well each model could predict the most suitable role for a task in a distributed agile team, where effective role assignment is critical for team performance and project success. Random Forest was chosen for its robustness in handling large feature sets, such as those generated from textual data, and its ability to capture the interactions between various features that could be important in determining role suitability. AdaBoost was incorporated into the study for its strength in improving weak learners and focusing on hard classification tasks, which is essential in a dynamic environment where some tasks may not have clear role assignments. The Decision Tree classifier was included for its interpretability, as it allows for an easy decision-making process. Lastly, K-NN was used due to its capability to make predictions based on the similarity of tasks, which fits well with the collaborative nature of agile teams where past task-role associations can guide future assignments.

By applying these models, our research aims to provide insights into the effectiveness of various ML approaches in predicting roles in DASD environments, offering a comparative analysis that could inform more efficient role allocation strategies in similar team structures. The main steps involved in conducting the research includes:

1. Dataset selection.
2. Data preprocessing, cleaning, and encoding of categorical features.
3. Developing the classification model.
4. Dataset splitting.
5. Model training, validation, and testing.
6. Model evaluation.

By following these steps, we gain knowledge about prediction job roles and matching employees. This will greatly contribute to improving the efficiency of task distribution and decision making in software management. Each of the steps is discussed as follows.

STEP1 Dataset selection

**Fig. 1:**
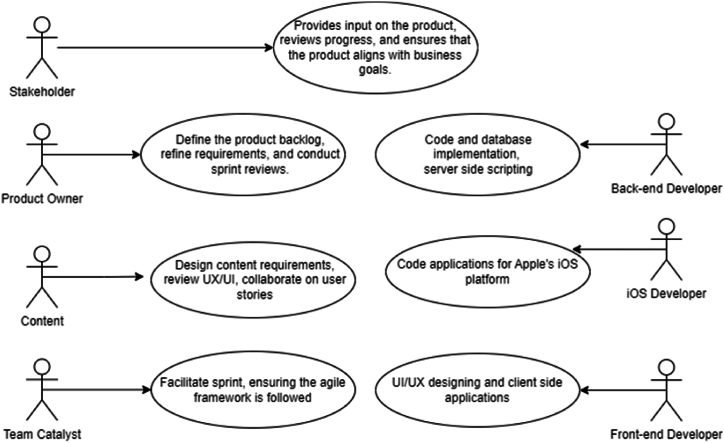
Description of roles in the dataset.

In this study, we obtained the dataset provided by Ref. [29] which is based on data extracted from the open access repository of agile teams “Taiga.io” that has active real project, tasks, team members, stories, sprints, and roles for every task. The features of the dataset encapsulate a wide array of tasks-centric information, empowering the analytical exploration of task allocation patterns, tendencies, and determinants. The dataset comprises a comprehensive set of 10 distinct projects of 1216 records (Project_ID; ProjectName; Title; Description; Role) with no missing data. The Project ID for the ten projects is (125501; 129529; 182862; 221277; 221716; 289231; 52931; 66937; 90369; 93128) which corresponds to the project names (Websites Durable Team; MDN Durable Team; Admin WebApp; Tymly; software-factory; TripleO CI (production); Distll; OpenSwitch; Eazycom; UXBOX) respectively. The titles of tasks (Title) from all the ten projects were broken down into individual words and transformed into sequences of numbers, preserving the structure and meaning between the words. A vocabulary index was created, ranking the words according to how often they appeared. These numerical representations were later used as input features for the learning model. The ML models are trained using the textual characteristics of past task assignments and predict the most suitable role (“Front-end Developer”, “Back-end Developer”, “iOS Developer”, “Product Owner”, “Team Catalyst”, “Content”, “Stakeholder”) for new tasks. A brief description of each role is described in Fig. 1.

STEP2 Data preprocessing, cleaning, and encoding of categorical features

**Fig. 2:**
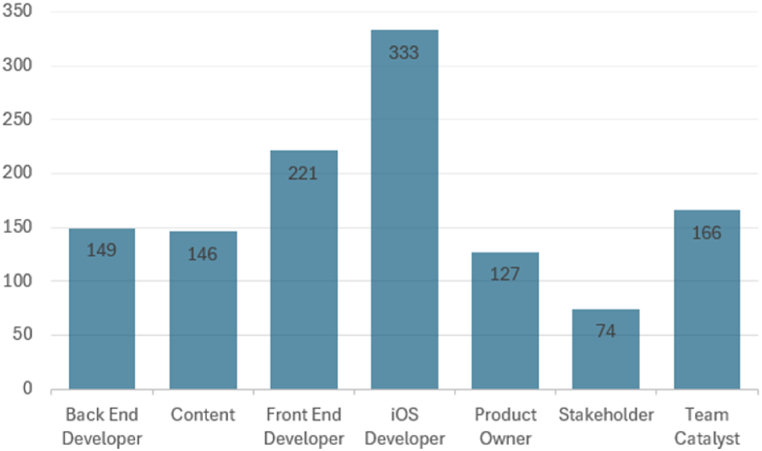
Imbalanced distribution of “Role” in the dataset.

In this study, data preprocessing plays a pivotal role in enhancing the performance of the machine learning model. This phase encompasses critical steps, including normalization, cleaning, and encoding of categorical data. Prior to these preprocessing procedures, an analysis of the dataset was conducted. Analyzing our dataset revealed an imbalanced distribution of class labels (Fig. 2) that can adversely affect the prediction of job roles by introducing biases that skew the model's understanding of the relationships between features and roles. Therefore, the model may overemphasize these features in predicting the more frequent roles while neglecting other characteristics that define fewer common roles. This can result in lower accuracy for predicting minority roles, as the model might struggle to capture their unique patterns due to a lack of sufficient examples.

**Fig. 3:**
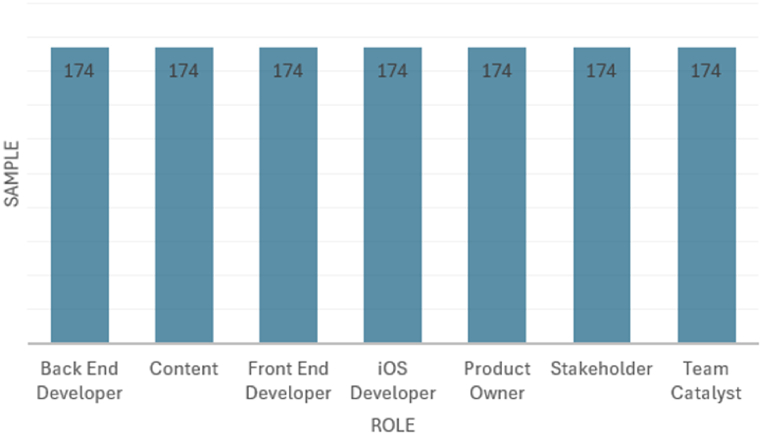
Balanced distribution of “Role” values.

To address the imbalanced distribution of classes, we employed the “SMOTETomek” technique which combines two methods: the Synthetic Minority Oversampling Technique (SMOTE) and Tomek links [4]. SMOTE generates additional samples to better represent the minority class in the dataset, while Tomek links help refine this process by identifying closely located. instances from different classes that may cause noise or misclassification. By creating new instances. that contain the key features of the minority class, SMOTE addresses class imbalance and clarifies boundaries between classes. Meanwhile, Tomek links remove majority class instances that are too close to minority class instances, enhancing overall class separation. The balanced classes obtained from this process are shown in Fig. 3.

The dataset contains categorical features such as Role, wherein the values belong to distinct groups rather than numeric scales. For effective utilization in most machine learning techniques, categorical features necessitate suitable encoding. These features must be converted to numeric values to be suitable for model training. To achieve this, the “One Hot Encoding” technique was employed, wherein a binary vector is allocated for each value of the categorical feature. For instance, if Role takes seven values, namely “Front-end Developer”, “Back-end Developer”, “iOS Developer”, “Product Owner”, “Team Catalyst”, “Content”, and “Stakeholder”, they would be mapped to binary vectors (1,0,0,0,0,0,0), (0,1,0,0,0,0,0), (0,0,1,0,0,0,0), (0,0,0,1,0,0,0), (0,0,0,0,1,0,0), (0,0,0,0,0,1,0), and (0,0,0,0,0,0,1) respectively.

Furthermore, the dataset exhibits significant variation in the range of feature values, potentially leading to reduced prediction accuracy. Features with larger values may disproportionately influence the model's performance compared to others [4]. To address this issue and ensure balanced data, normalization is employed. Normalization rescales the feature values to a specific range, mitigating the impact of disparate scales on the ML model.

By conducting data preprocessing, cleaning, and categorical feature encoding, we aim to optimize the model's performance and enhance accurate predictions in our study. These preparatory steps are crucial for training the classification model and enhancing the model's accuracy.

STEP3 Developing the classification model

Orange Data Mining Tool (Orange 3.36.1) has been utilized for developing the classification model [30]. It is a comprehensive and open-source software equipped with a wide array of standard and advanced machine learning and data mining algorithms. Its user-friendly interface empowers users to perform data visualization, analysis, and model building. The tool is particularly suitable for handling substantial datasets. At its core, Orange Data Mining Tool is a C++ library complemented by a collection of Python-based modules, fostering a scriptable environment for quick algorithm prototyping and pattern testing. It efficiently implements various functionalities, prioritizing ease of use and convenience over execution time [30].

Furthermore, Orange widgets provide a graphical user interface that simplifies access widgets, including those for data entry, preprocessing, classification, regression, association rules, clustering, and model assessment. This approach allows us to create a robust prediction model while harnessing the versatility of the Orange Data Mining Tool. The widgets also facilitate visualizing assessment results, enhancing the overall user experience and promoting effective model evaluation [30].

**Fig. 4:**
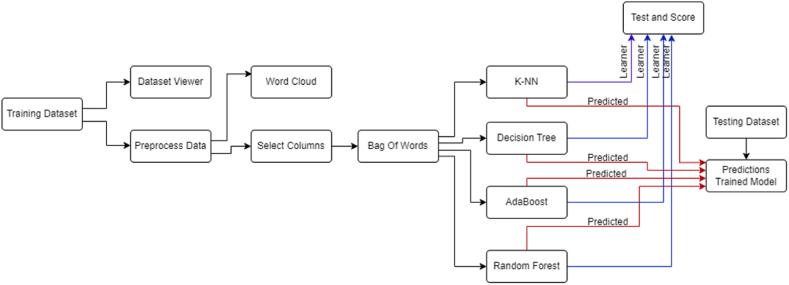
The research model.

The developed model is showcased in Fig. 4, where the model utilizes a widget called corpus to receive and preprocess the dataset through tokenization and filtering. The dataset is then split into two halves: one for training the ML algorithms and the other for making predictions with the model.

In our methodology, we initially started with the default base parameters for each of the classifiers (AdaBoost, Random Forest, Decision Tree, and K-NN) to establish a baseline for performance. These default values are commonly used and provide a standard starting point for model evaluation [6]. After obtaining initial results, we proceeded to fine-tune the parameters to enhance the performance of each classifier [31]. Table 1 shows both the default values and the adjusted (tuned) values that were used to improve the models' predictive accuracy and generalization. By adjusting parameters such as the number of estimators, learning rates, tree depth, and neighbors, we were able to optimize the classifiers for better results.

STEP4 Dataset splitting

**Table 1:** Parameters for AdaBoost, Random forest, decision tree, and K-NN.

Classifier	Parameter	Default Value	Tuned Value	Description
AdaBoost	n_estimators	50	100	We increased the number of weak learners for better accuracy
	learning_rate	1.0	0.5	We reduced the contribution of each classifier for smoother learning
	Algorithm	‘SAMME.R′	‘SAMME.R′	We kept the real boosting algorithm for more stable performance
Random Forest	n_estimators	100	200	We increased the number of trees for better model generalization
	Criterion	“gini"	“entropy"	We used entropy for better splits
	max_depth	–	20	We limited the depth to 20 to prevent overfitting while maintaining good accuracy
	min_samples_split	2	4	The min samples split was increased to 4 to prevents overfitting by requiring more samples to split a node
Decision Tree	Criterion	“gini"	“entropy"	We used entropy for better splits
	max_depth	–	10	We set the depth to 10 to ensure a moderate tree depth and avoid overfitting
	min_samples_split	2	5	We increased the samples to split the nodes to 5 for smoother tree structure
K-NN	n_neighbors	5	10	We increased the number of neighbors to 10 for better generalization
	Algorithm	auto	Auto	The automatic algorithm selection has been set to ‘auto’ based on the data size
	Metric	‘minkowski’ with p = 2 (Euclidean Distance)	‘minkowski’ with p = 2 (Euclidean Distance)	Euclidean distance with p = 2 has been used for continuous features

In this phase, the dataset is strategically split into three subsets: training, validation, and testing sets. The purpose of this division is to enable effective model evaluation. We used the training dataset to teach the models, then we tuned and optimized the model parameters using the validation dataset. After that, we assessed the performance of the trained models using the test dataset. This approach ensures that our models are reliable and can be applied to situations and draw conclusions about their predictive abilities.

STEP5 Model training, validation, and testing

The developed models went through a training and validation process using the dataset. We use the training dataset to teach the models how to recognize patterns and relationships in the data. The trained model then analyzes the dataset to classify data and make predictions. During the training process, we optimized and tuned the models' parameters using the validation subset and evaluated their ability to generalize and assessed how well they handle situations. The model is assessed using K-fold cross-validation, “a technique that evaluates its generalizability by dividing the dataset into K-parts” [20]. It is trained on K-1 folds, tested on the last fold, and repeated K-times. Once parameters are optimized, the best-performing model from the cross-validation process is chosen.

After the training process, we subject the ML models to testing using the test dataset that has not been seen before. We meticulously evaluated the accuracy of their predictions using the 80 20 rules; 80 % of our dataset is used for training while 20 % is reserved for testing. The results from this testing phase are crucial to assign roles for a given task based on those predictions.

STEP6 Performance evaluation

Evaluating the performance of ML algorithms is crucial to understand how effective and accurate they are [32,33]. Using metrics, like accuracy, precision, recall and F-measure, we can gain insights into how well the model predicts outcomes and handles various classes. A brief discussion of the metrics used to evaluate the performance of our proposed model is given as follows.

A. Accuracy:

Accuracy (ACC) is a metric that measures how often the results are correctly represented as a percentage, to the number of cases examined [33]. It is rated on a scale from 0 to 1 where 1 signifies accuracy. The formula to calculate accuracy is as follows [12];

(1) ACC = (TP + TN) / (TP + TN + FP + FN)

B. Precision (Positive Predictive Value):

Precision measures the number of predictions in relation to the overall number of positive predictions made. This value can be calculated using the formula [33];

(2) Precision = TP / (TP + FP)

C. Recall (True Positive Rate or Sensitivity):

Recall, also known as True Positive Rate or Sensitivity calculates the number of positive predictions in relation to the total number of actual positives [33]. It ranges from 0 to 1 with 1 indicating recall. The formula for calculating recall is [33];

(3) Recall = TP / (TP + FN)

D. F-Measure:

The F-measure combines precision and recall into an evaluation criterion by using their mean [33]. This balanced metric is commonly employed for comparison, between machine learning algorithms [4].

(4) F-measure = (2 ∗ Recall ∗ Precision) / (Recall + Precision)

By utilizing these metrics for performance evaluation we can obtain insights, into the models ability to predict, allowing us to make decisions and comparisons between the various ML algorithms employed in this study.

## 5 Results

In this study, we employed four ML algorithms for the modeling process: Decision Tree, Random Forest, K-Nearest Neighbors (K-NN), and AdaBoost classifiers. The primary objective was to identify the most suitable classifier for predicting job roles. Each model was trained and tested using inputs from the datasets, with the data split into 80 % for training and 20 % for testing. The results of the evaluations for all models are presented in Table 2.

**Table 2:** The results obtained from testing the four-machine learning models.

Model	Accuracy	AUC	F1	Precision	Recall
Decision Tree	0.935	0.762	0.745	0.746	0.762
Random Forest	**0.967**	0.786	0.746	0.745	0.786
K-NN	0.942	0.769	0.726	0.750	0.769
AdaBoost	0.930	0.784	0.775	0.773	0.784

Each model's performance is evaluated using metrics, including AUC, Accuracy, F1-score, Precision, and Recall. These metrics offer insights into how the models predict and classify job roles. As shown in Table 2, the Random Forest classifier achieved the AUC score of 0.762, which indicates its ability to distinguish between positive and negative instances. The Accuracy of the Random Forest model stands at 0.967 meaning it accurately predicted job roles for around 97 % of instances in the dataset.

The Decision Tree classifier also performed well with an AUC score of 0.762 and an Accuracy score of 0.935, while slightly lower than the Random Forest classifier, it still demonstrates good performance. The K-NN classifier achieved an AUC score of 0.769 and an Accuracy score of 0.942. Its F1-score of 0.726 suggests that it may not capture the balance between Precision and Recall as effectively. Lastly, we have the AdaBoost classifier with an AUC score of 0.784 and an Accuracy of 0.930. It shows an F1-score of 0.775 indicating a balance between Precision and Recall.

**Fig. 5:**
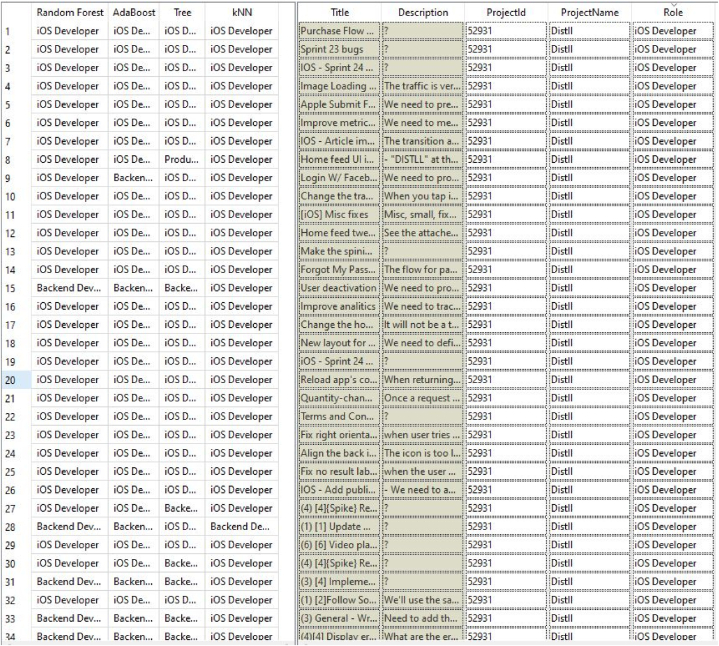
Actual Role vs Predicted Role.

**Fig. 6:**
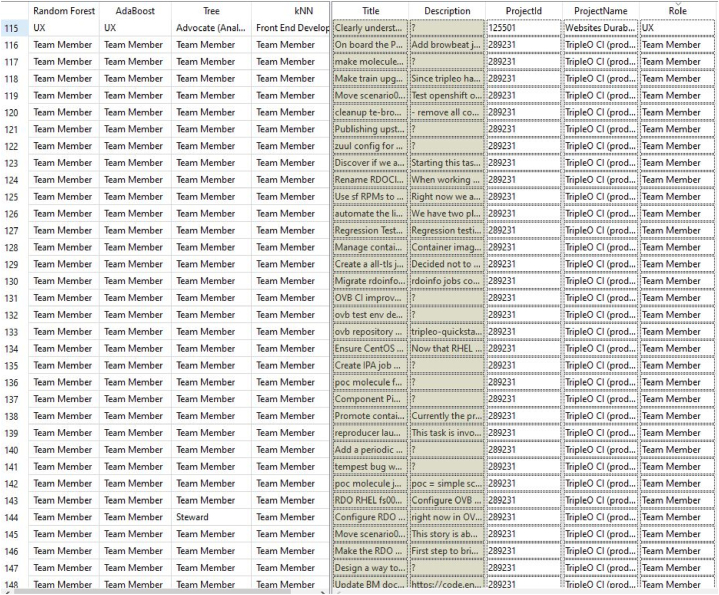
Actual Role vs Predicted Role.

Based on the results of the conducted experiments, we found that the Random Forest classifier outperforms tested algorithms in terms of accuracy when predicting job roles. It shows promising results for real world applications. Fig. 5, Fig. 6 present a comparison between the job roles of employees and the roles predicted by machine learning algorithms. The actual role “Role” column represents the roles of employees while the predicted Role utilizing different ML algorithms column displays the roles predicted by the four employed ML models. This comparison allows us to evaluate how accurately these algorithms identify employees’ roles and their effectiveness overall.

## 6 Discussion of results

This research focuses on analyzing assigned tasks of different projects derived from a real dataset, aiming to predict a suitable role for a new task. It employs ML algorithms, such as (Decision Tree, Random Forest, K-NN, AdaBoost) to forecast the role for a given task within agile environments. The titles are transformed into sequence vectors, which are then utilized as multidimensional features for training the ML classifiers. We classify roles into seven generic classes based on the team roles with comparable descriptions obtained from the dataset.

The results obtained from testing the four machine learning models, as summarized in Table 2, provide insightful observations regarding their performance in predicting task allocations within software development teams. Among the models evaluated, the Random Forest algorithm demonstrated the highest accuracy of 96.7 % and an AUC score of 0.786. This indicates that Random Forest not only made accurate predictions but also effectively distinguished between the different roles suitable for various tasks. The precision and recall values of 0.745 and 0.786, respectively, suggest that while the model maintained a good balance between correctly predicting relevant roles and capturing the actual instances of those roles, there is room for improvement, particularly in minimizing false positives.

In contrast, the Decision Tree model, while exhibiting a commendable accuracy of 93.5 %, had lower values in AUC (0.762) and F1 score (0.745). This suggests that the Decision Tree model may be less reliable in discriminating between suitable roles, which could result in suboptimal task allocations. The relatively lower performance of the Decision Tree could be attributed to its propensity to overfit the training data, highlighting a common limitation in simpler models.

The K-Nearest Neighbors (K-NN) model achieved an accuracy of 94.2 %, with a notable AUC of 0.769. Its precision of 0.750 and recall of 0.769 demonstrate its capability in maintaining a reasonable balance between correctly identifying relevant roles and ensuring a comprehensive capture of all instances. However, its slightly lower accuracy compared to Random Forest suggests that while K-NN can be useful in specific scenarios, it may not perform as robustly in all contexts.

The AdaBoost algorithm, with an accuracy of 93.0 % and an AUC of 0.784, provided comparable performance to the other models but fell short in precision (0.773) and recall (0.784). This performance indicates that while AdaBoost can enhance weaker classifiers, its effectiveness may be limited in the context of this specific task allocation problem.

In summary, the results indicate that ML algorithms used in our research achieved a remarkable accuracy rate of 96.7 % by Random Forest classifier. This implies that approximately 97 % of the predictions made by this algorithm are accurate. When comparing these results with existing literature, it is evident that methods like Random Forest generally outperform single classifiers, aligning with findings from prior studies of [4,6,12], and [21]. This emphasizes the importance of model selection in achieving high predictive accuracy. However, it is crucial to acknowledge the challenges faced in this research area, particularly the scarcity of publicly available datasets. Information regarding team member roles is often not accessible to external researchers, limiting the ability to conduct comparative studies. Notably, we are unaware of any studies that have utilized machine learning algorithms specifically for task allocation within software development teams. The only relevant study we identified, which employed the same dataset as ours is the study of [20] which achieved a maximum accuracy of 69 %. In contrast, our research attained an impressive accuracy of 96.7 %, highlighting the effectiveness of our approach.

The implications of these findings provide valuable insights for researchers by demonstrating how the textual attributes of tasks can be used as reliable predictors utilizing ML algorithms for identifying variety of roles within agile teams. Additionally, project managers in software development environments, as organizations, can improve their decision-making processes regarding task allocation, potentially boosting team productivity and project outcomes.

## 7 Conclusion and future work

In this study, we aim to tackle the hurdles of assigning tasks in agile software development environments. Assigning tasks is the responsibility of project managers in teams as they must carefully evaluate various factors before allocating tasks to team members. To tackle this challenge, we employed ML models to forecast the allocation of job roles for a given task among employees. Our focus was on task allocation within DASD settings, where geographical distance makes adherence to agile principles harder. The results show that ML models are capable of properly predicting task assignments. Notably, in this prediction task, the Random Forest classifier outperformed other algorithms with accuracy of 96.7 %.

The research shows that ML models can address task allocation challenges in remote agile software development environments. However, it is important to acknowledge the limitations of our study, including potential bias in the dataset and the restricted variety of models examined. For future research, we recommend exploring additional ML models, such as gradient boosting or deep learning approaches, which may provide further improvements in accuracy and predictive capabilities. Additionally, incorporating more diverse datasets and conducting feature importance analysis could yield insights into which factors most significantly influence task allocation decisions, ultimately enriching the model's performance and applicability in real-world scenarios.

## CRediT authorship contribution statement

**Dimah Al-Fraihat:** Writing – review & editing, Writing – original draft, Supervision, Software, Project administration, Methodology, Formal analysis. **Yousef Sharrab:** Writing – review & editing, Writing – original draft. **Abdel-Rahman Al-Ghuwairi:** Resources, Investigation. **Hamza Alzabut:** Software, Formal analysis. **Malik Beshara:** Software, Formal analysis. **Abdulmohsen Algarni:** Writing – review & editing, Funding acquisition.

## Ethics approval and consent to participate

Not applicable.

## Data availability statement

The data presented in this study is available and can be accessed at https://github.com/jku-isse/TaskAllocator/blob/master/Dataset/125501.csv.

## Funding

This research was financially supported by the 10.13039/501100023674Deanship of Scientific Research at King Khalid University under research grant number (R.G.P.2/93/45).

## Declaration of competing interest

The authors declare that they have no known competing financial interests or personal relationships that could have appeared to influence the work reported in this paper.

## References

[1] Mohapatra H., Rath A.K. (2020).

[2] Singh M., Chauhan N., Popli R. (2020, May). Proceedings of the International Conference on Innovative Computing & Communications (ICICC).

[3] Alhazmi A., Huang S. (2020, March). 2020 SoutheastCon.

[4] Al-Fraihat Dimah, Sharrab Yousef, Al-Ghuwairi Abdel-Rahman, AlElaimat Majed, Alzaidi Maram (2024). Detecting and resolving feature envy through automated machine learning and move method refactoring. Int. J. Electr. Comput. Eng..

[5] Paetsch F., Eberlein A., Maurer F. (2003, June). WET ICE 2003. Proceedings. Twelfth IEEE International Workshops on Enabling Technologies: Infrastructure for Collaborative Enterprises, 2003.

[6] Al-Fraihat D., Sharrab Y., Al-Ghuwairi A.R., Alshishani H., Algarni A. (2024). Hyperparameter optimization for software bug prediction using ensemble learning. IEEE Access.

[7] Kafetzis D., Vassilaras S., Vardoulias G., Koutsopoulos I. (2022). Software-defined networking meets software-defined radio in mobile ad hoc networks: state of the art and future directions. IEEE Access.

[8] Fatima T., Azam F., Anwar M.W., Rasheed Y. (2020, April). Proceedings of the 2020 6th International Conference on Computing and Artificial Intelligence.

[9] Al-Ghuwairi A.R., Al-Fraihat D., Sharrab Y., Alrashidi H., Almujally N., Kittaneh A., Ali A. (2023). Visualizing software refactoring using radar charts. Sci. Rep..

[10] Ali K., Alzaidi M., Al-Fraihat D., Elamir A.M. (2023).

[11] Al-Fraihat D., Sharrab Y., Alzyoud F., Qahmash A., Tarawneh M., Maaita A. (2024). Speech recognition utilizing deep learning: a systematic review of the latest developments. Human-centric Computing and Information Sciences.

[12] Al-Fraihat D., Sharrab Y., Al-Ghuwairi A.R. (2024). Detecting refactoring type of software commit messages based on ensemble machine learning algorithms. Sci. Rep..

[13] Aslam W., Ijaz F. (2018). A quantitative framework for task allocation in distributed agile software development. IEEE Access.

[14] Yadav A., Singh S.K., Suri J.S. (2019). Ranking of software developers based on expertise score for bug triaging. Inf. Software Technol..

[15] Anvik J., Hiew L., Murphy G.C. (2006, May). Proceedings of the 28th International Conference on Software Engineering.

[16] Nundlall C., Nagowah S.D. (2021). Task allocation and coordination in distributed agile software development: a systematic review. Int. J. Inf. Technol..

[17] Aslam W., Ijaz F. (2018). A quantitative framework for task allocation in distributed agile software development. IEEE Access.

[18] Ijaz F., Aslam W., AlSanad A.A., Aslam Z., Ullah I. (2022).

[19] Nundlall C., Nagowah S.D. (2022). Task allocation and coordination process in distributed agile software development: an ontology based approach. Inf. Technol. Manag..

[20] Shafiq S., Mashkoor A., Mayr-Dorn C., Egyed A. (2021, May). 2021 IEEE/ACM Joint 15th International Conference on Software and System Processes (ICSSP) and 16th ACM/IEEE International Conference on Global Software Engineering (ICGSE).

[21] William P., Kumar P., Chhabra G.S., Vengatesan K. (2021, November).

[22] Singh M., Chauhan N., Popli R. (2020). Machine learning based backlog prioritization techniques in distributed agile software development. International Journal of Advanced Science and Technology.

[23] Hammouri A., Hammad M., Alnabhan M., Alsarayrah F. (2018). Software bug prediction using machine learning approach. Int. J. Adv. Comput. Sci. Appl..

[24] Belgiu M., Drăguţ L. (2016). Random forest in remote sensing: a review of applications and future directions. ISPRS J. Photogrammetry Remote Sens..

[25] Mienye I.D., Sun Y. (2022). A survey of ensemble learning: concepts, algorithms, applications, and prospects. IEEE Access.

[26] Song Y.Y., Ying L.U. (2015). Decision tree methods: applications for classification and prediction. Shanghai archives of psychiatry.

[27] Peterson L.E. (2009). K-nearest neighbor. Scholarpedia.

[28] Anvik J., Murphy G.C. (2011). Reducing the effort of bug report triage: recommenders for development-oriented decisions. ACM Trans. Software Eng. Methodol..

[29] Shafiq S. (2017). TaskAllocator dataset. https://github.com/jku-isse/TaskAllocator/blob/master/Dataset/125501.csv.

[30] Demšar J., Curk T., Erjavec A., Gorup Č., Hočevar T., Milutinovič M., Zupan B. (2013). Orange: data mining toolbox in Python. the Journal of machine Learning research.

[31] Yin M., Wortman Vaughan J., Wallach H. (2019, May). Proceedings of the 2019 Chi Conference on Human Factors in Computing Systems.

[32] Sharrab Y., Al-Fraihat D., Tarawneh M., Sharieh A. (2023, June). 2023 International Conference on Multimedia Computing, Networking and Applications (MCNA).

[33] Tharwat A. (2021). Classification assessment methods. Appl. Comput. Inform..

